# Doxycycline impairs neutrophil migration to the airspaces of the lung in mice exposed to intratracheal lipopolysaccharide

**DOI:** 10.1186/1476-9255-9-31

**Published:** 2012-09-03

**Authors:** Andres Moon, Sucheol Gil, Sean E Gill, Peter Chen, Gustavo Matute-Bello

**Affiliations:** 1 The Center for Lung Biology, Division of Pulmonary & Critical Care Medicine, Department of Medicine, University of Washington School of Medicine, 850 Republican Street, Box 358052, Seattle, 98109-4714, , WA, USA; 2 The Centre for Critical Illness Research, Lawson Health Research Institute, Department of Medicine, Western University, London, ON, Canada

**Keywords:** Doxycycline neutrophils lipopolysaccharide inflammation

## Abstract

**Background:**

Tetracyclines are broad-spectrum antibiotics that are also used to induce gene expression using the reverse tetracycline transactivator / tetracycline operator system (rtTA/tetO system). The system assumes that tetracyclines have no effects on mammals. However, a number of studies suggest that tetracyclines may have powerful anti-inflammatory effects. We report that the tetracycline, doxycycline, inhibits neutrophil (PMN) influx into the lungs of mice treated with bacterial endotoxin (LPS).

**Methods:**

Mice were challenged with intratracheal LPS in the presence or absence of doxycyline. bronchoalveolar lavage cell counts and differential, total bronchoalveolar lavage protein, lung homogenate caspase-3 and tissue imaging were used to assess lung injury. In addition, PMN chemotaxis was measured *in vitro* and syndecan-1 was measured in bronchoalveolar lavage fluid.

**Results:**

The administration of doxycycline resulted in a significant decrease in the number of bronchoalveolar lavage PMNs in LPS-treated mice. Doxycycline had no effect on other markers of lung injury such as total bronchoalveolar lavage protein and whole lung caspase-3 activity. However, doxycycline resulted in a decrease in shed syndecan-1 in bronchoalveolar lavage fluid.

**Conclusion:**

We conclude that doxycycline has an important anti-inflammatory effect that can potentially confound the experiments in which the rtTA/tetO system is being used to study the immune response.

## Background

One of the most important methods to investigate mechanisms of disease is the deletion of specific genes to perform loss-of-function experiments. However, traditional “knockout” mice, in which a gene is deleted from the onset of embryonic development, may respond to the loss of the gene by developing compensatory mechanisms that can confound the results of experiments. In 1992, Gossen and Bujard developed a system to activate genes in a time-specific manner—in essence, an “on/off” switch— [1]. The system consists of two elements: the first is the tetracyclin-responsive element (TRE), which is composed of the *E. coli tet* operator (tetO) fused with a minimal promoter sequence from the CMV virus. The second element is the tetracyclin-controlled transactivator tTA, created by fusing the *E. coli* tet repressor with the activating domain of virion protein 16 of herpes simplex virus. The key aspect of the system is that TRE only initiates transcription when bound to tTA, and the binding of tTA to TRE is dependent on the presence or absence of a tetracycline. There are two versions of the system: in the “tet-off” version of the system, tTA binds to TRE exclusively in the absence of a tetracycline; in the “tet-on” version of the system, a reverse tTA (rtTA) binds to TRE exclusively in the presence of a tetracycline (reviewed in [2]). By placing the tTA/rtTA sequence downstream from a tissue-specific promoter; and the TRE proximal to a sequence of interest, a tissue specific, inducible gene expression system is generated. Commonly, doxycycline is used as the tetracycline of choice, and the entire system assumes that doxycycline has no effect of its own on the responses of the model being studied.

Doxycycline is a member of the tetracycline family of broad spectrum antibiotics (reviewed in [3]). Discovered in the 1940s, this family is characterized by molecules composed of a linear fused tetracyclic nucleus to which a number of functional groups have been attached [3]. The antibiotic properties of tetracyclines are based on high affinity binding with the bacterial 30S ribosomal units to inhibit protein synthesis. Importantly, tetracyclines have very poor affinity for eukaryotic ribosomes, and for this reason have relatively few side effects in eukaryotes. However, some reports suggest that tetracyclines may have an anti-inflammatory effect in the lungs. For example, Fujita *et al.* have shown that doxycycline attenuates PMN recruitment in models of lung injury secondary to LPS, bleomycin or *Streptococcus pneumoniae* pneumonia, and speculate that this is due to inhibition of metalloproteinases [4,5]. If confirmed, these findings could have important implications for models using tetracyclines to induce gene expression.

We recently used the rtTA/tetO system to investigate the role of the adapter protein FADD in LPS-induced lung injury. In the course of the experiments we also found a very clear and profound anti-inflammatory effect of doxycyline. We are publishing these results to illustrate the potential confounding effects of doxycyline and the need for strict controls in experiments involving the rtTA/tetO system.

## Methods

### Animal protocol

All of the animal experiments were approved by the Institutional Animal Care Committee of the University of Washington. Mice carrying the reverse tetracycline transactivator under control of the epithelial cell promoter CCSP (CCSP-rtTA) were kindly provided by Jeffrey Whitsett. Seventy-two hours prior to the experiments, some of the mice received doxycycline, 2 mg/mL in the drinking water. On the day of the experiments, the mice where anesthetized with inhaled isoflurane, intubated endotracheally with a 20-ga catheter, and given intratracheal installations of either PBS or LPS, 150 ng/gram. The instillate was suspended in 2.5% colloidal carbon to allow later confirmation of the extent and distribution of the instillation macro and microscopically. The mice were returned to their cages for 24 h, at which point they where euthanized with intraperitoneal injections of Beuthanasia D. The thorax was rapidly opened, the left hilum was clamped, sutured, and the left lung was removed and flash frozen in liquid nitrogen. The right lung was lavaged with three 0.5 mL aliquots of PBS containing 0.6 mM EDTA (PBS/EDTA) and then fixed in 4% paraformaldehyde at an inflation pressure of 15 cm of water.

### Experimental design

The following groups of mice were studied: Mice treated with PBS only (n = 3), mice treated with doxycycline only (n = 5), mice treated with LPS only (n = 5), and mice treated with the combination of LPS and doxycycline (n = 6).

### Sample processing

BAL cell counts were performed on a hemacytometer. Differentials were performed on cytospin preparations stained with a modified Wright-Giemsa stain (Diff-Quick, Fischer Scientific Company, Kalamazoo, MI). Total proteins were measured with the bicinchoninic acid method (BCA assay, Pierce, Rockford, IL). BAL IgM was measured using a specific ELISA (Bethyl Laboratories, Montgomery, TX).

### PMN chemotaxis

C57BL/6 mice were euthanized by exposure to CO_2_ followed by cervical dislocation. The femur and tibia of both hind legs were isolated and freed of all soft tissue, and then the ends of both bones were removed. The femur and tibia were then placed proximal end down in a 0.6 mL Eppendorf tube, which had been punctured at its lower tip with an 18-gauge needle and placed inside a 1.5 mL Eppendorf tube. The tubes were spun at 2000 x *g* for 30 s and PMNs were then isolated as previously described [6,7]. After isolation, PMNs were incubated for 60 min in RPMI containing 1% BSA, calcein-AM (5 μg/ml; Molecular Probes, Eugene, OR) and PBS, 0.5, 1.0, or 2.0 μg/mL Doxycycline. PMNs were then washed two times in phosphate buffered saline (PBS) and resuspended at a concentration of 1 × 10^6^/mL. PMN chemotaxis towards KC (1–333 ng/mL) (PeproTech Inc., Rocky Hill, NJ) was then assessed using the Neuro Probe ChemoTx Disposable Chemotaxis system (Neuro Probe Inc. Gaithersburg, MD) with the Synergy 4 plate reader (BioTek, Winooski, VT).

### Immunoblots

We performed a dot blot for syndecan-1 as previously described [8]. In brief, dot blot buffer was added to 200 μl of BAL sample (final concentration: 150 mM NaCl, 50 mM NaOAc, 0.1% Triton-X 100; pH 4.5). Samples were loaded onto cationic Immobilon Ny + nylon membranes (Millipore) and immunoblotted with polyclonal rabbit anti-mouse syndecan-1 antibody (clone 281.2; 1:1000; BD Biosciences).

### Histology

Lung sections were embedded in paraffin, cut into 4 μm sections, and stained with hematoxylin and eosin. Cryosections were cut fresh, directly placed on the tissue slide, and immediately visualized in a fluorescence microscope. PMN were counted on 10 randomly generated high power fields using morphologic criteria (nuclear shape, color and size).

### Statistical methods

Statistical analysis was performed using ANOVA. When significant, the ANOVA was followed by the Bonferroni post hoc analysis. Analysis was performed with GraphPad Prism software. A p-value of less than 0.05 was considered significant.

## Results

**Figure 1:**
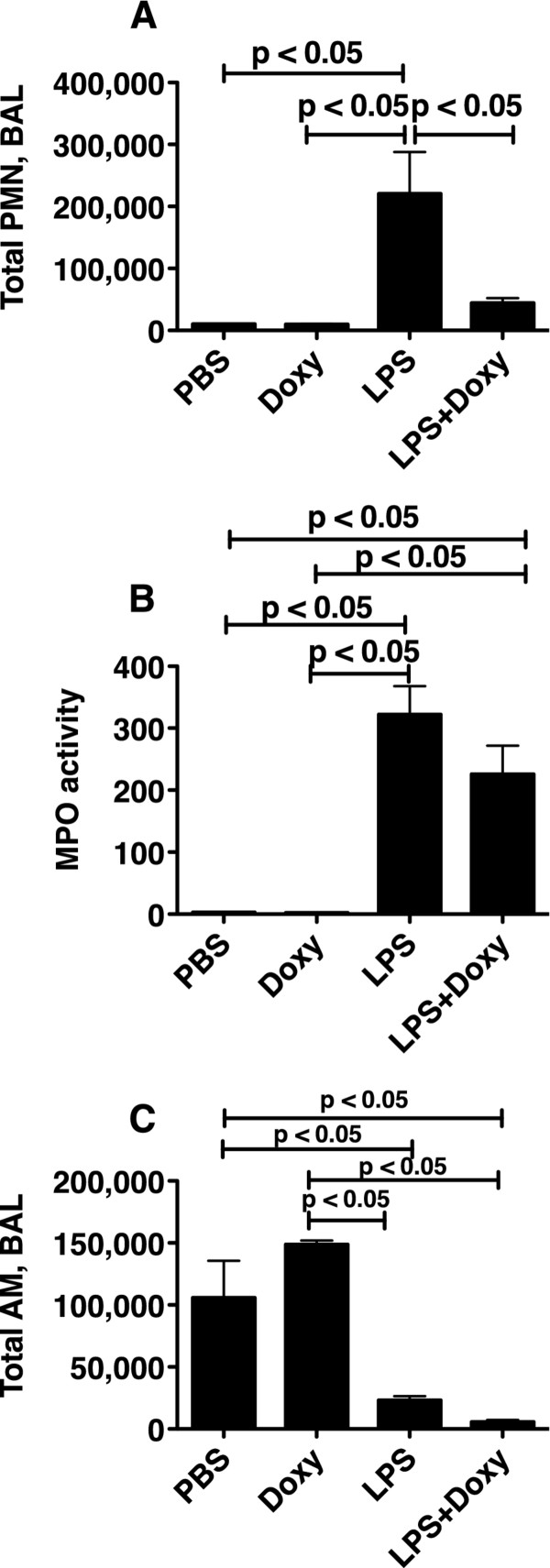
**Cellular responses.** Total BAL PMNs (**A**), lung homogenate MPO activity (**B**) and total BAL alveolar macrophages (AM) (**C**) in mice 24 h following the intratracheal administration of LPS (150 ng/gm) or PBS. Some of the mice received doxycycline, 2 mg/kg in the drinking water starting 72 h prior to the administration of LPS. The BAL PMN were increased only in the LPS-treated mice without doxy, whereas lung MPO activity was increased in LPS-treated mice with and without doxy. This suggests that neutrophils accumulated in the lungs, but did not enter the alveolar spaces. n: PBS = 3; Doxy = 5, LPS = 5, LPS + Doxy = 6.

The administration of intratracheal LPS to the lungs of mice was followed 24 h later by an increase in the total number of PMN in the BALF (Figure 1-A). However, when mice were given doxycycline prior to the administration of LPS, the neutrophilic response was significantly attenuated (p < 0.05). In contrast, lung MPO activity, indicative of the total content of PMN in the lungs, was increased in all of the LPS-treated mice, regardless of doxycycline (Figure 1-C). The number of BAL macrophages decreased in response to LPS administration, and remained low when doxycycline was given prior to LPS (Figure 1-C).

**Figure 2:**
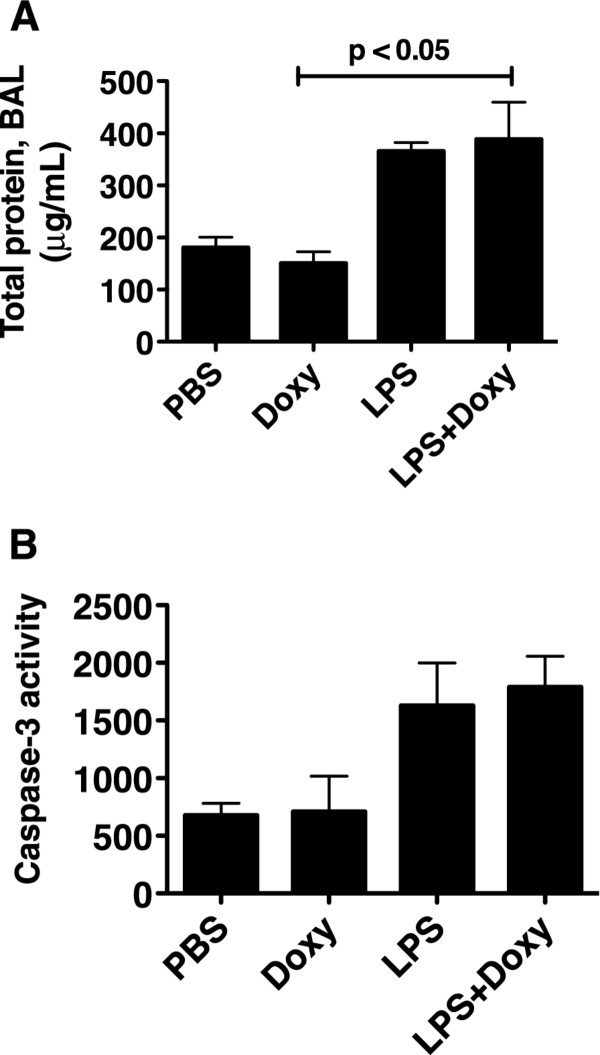
**Permeability and apoptotic responses.** Total BAL protein (**A**) and whole lung caspase-3 activity (**B**) in mice 24 h following the intratracheal administration of LPS (150 ng/gm) or PBS. Some of the mice received doxycycline, 2 mg/kg in the drinking water starting 72 h prior to the administration of LPS. LPS caused an increase in total BAL protein and in the lung caspase-3 activity, which was independent of doxy. n: PBS = 3; Doxy = 5, LPS = 5, LPS + Doxy = 6.

As an assessment of changes in the permeability of the alveolar-capillary barrier we measured the total protein concentration in the BALF. There was a modest increase in BALF total protein in the LPS-treated animals, which reached significance for the comparison “doxy only” vs “LPS + doxy” (Figure 2-A). The mice treated with LPS only had similar BALF total protein concentrations as the mice treated with LPS and doxy. We also measured caspase-3 activity as an indicator of apoptosis. There was a trend towards higher caspase-3 activity in the LPS-treated mice, which was unaffected by doxy (Figure 2-B).

**Figure 3:**
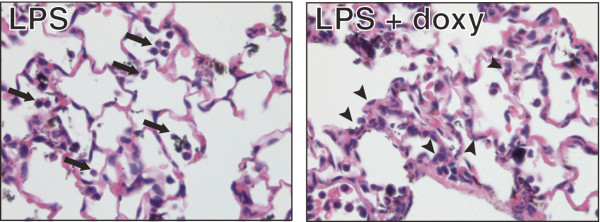
**Tissue responses.** Lung tissues from mice treated with intratracheal LPS in the presence or absence of oral doxycycline. The lungs of mice studied 24 h after the administration of intratracheal LPS showed intra-alveolar inflammatory infiltrates (arrows), which were absent in mice that received oral doxycycline starting 72 h prior to the administration of LPS. However, there were abundant neutrophils in the alveolar walls of the mice treated ith LPS and doxy (arrowheads). Note that in either case the alveolar walls remained thin.

To investigate the pattern of lung injury we evaluated the tissue response of animals treated with LPS in the presence or absence of doxy. Histologically, animals treated with LPS without doxy showed intra-alveolar neutrophilic infiltrates, which were absent in the presence of doxy (Figure 3). The thickness of the alveolar walls was increased in all animals treated with LPS, regardless of the presence of doxycycline. In the mice treated with LPS and doxycycline, fewer PMNs infiltrated the airspaces than in mice treated with LPS alone, but abundant PMNs remained in the alveolar walls (arrowheads, Figure 3) and blood vessels. The average number of intra-alveolar PMN per 400X field was 14 ± 1.9 in the LPS group, vs 2 ± 0.6 in the LPS + Doxy group.

**Figure 4:**
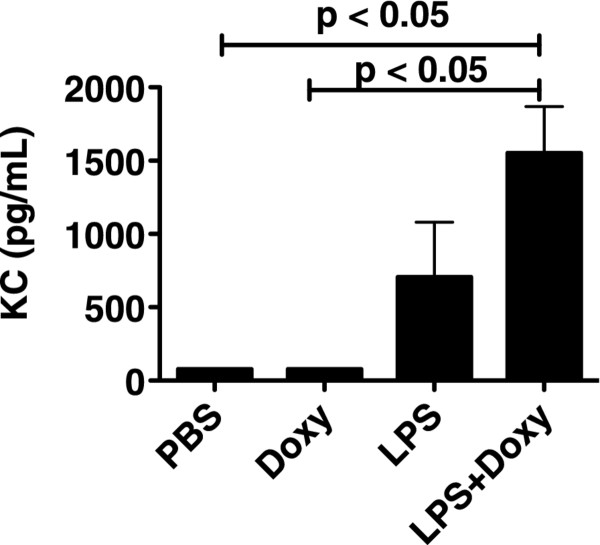
**KC concentrations.** BAL concentrations of KC in mice studied 24 h after the administration of LPS (150 ng/g) or PBS, in the presence or absence of doxycycline, 2 mg/kg in drinking water starting 72 h prior to the administration of LPS. There was a significant increase in KC concentrations in the mice treated with LPS and doxy. n: PBS = 3; Doxy = 5, LPS = 5, LPS + Doxy = 6.

**Figure 5:**
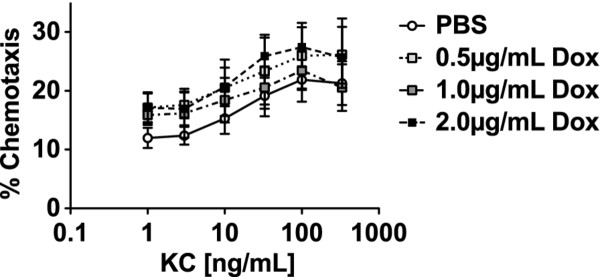
**PMN chemotaxis.** Percentage of murine bone marrow PMNs migrating in response to serial concentrations of KC in the presence of either PBS or 3 different concentrations of doxycycline. Treatment with doxy did not impair neutrophil chemotaxis to KC *in vitro*. n = 3.

PMNs enter the airspaces of the lung in response to chemotactic gradients, and one of the most important PMN chemoattractants in mice is the chemokine KC (CXCL1). Thus, we next investigated the lung BAL concentrations of KC. Interestingly, the highest concentrations of KC were seen in the mice treated with LPS and doxy (Figure 4). We next tested whether doxy impairs murine PMN chemotaxis against the KC. Murine PMNs were incubated with three different concentrations of doxy or PBS, and their chemotaxis towards KC was measured in a chemotactic chamber. There was no significant difference in PMN chemotaxis towards KC at all of the doxy doses tested, as compared with PMNs incubated with PBS (Figure 5).

**Figure 6:**
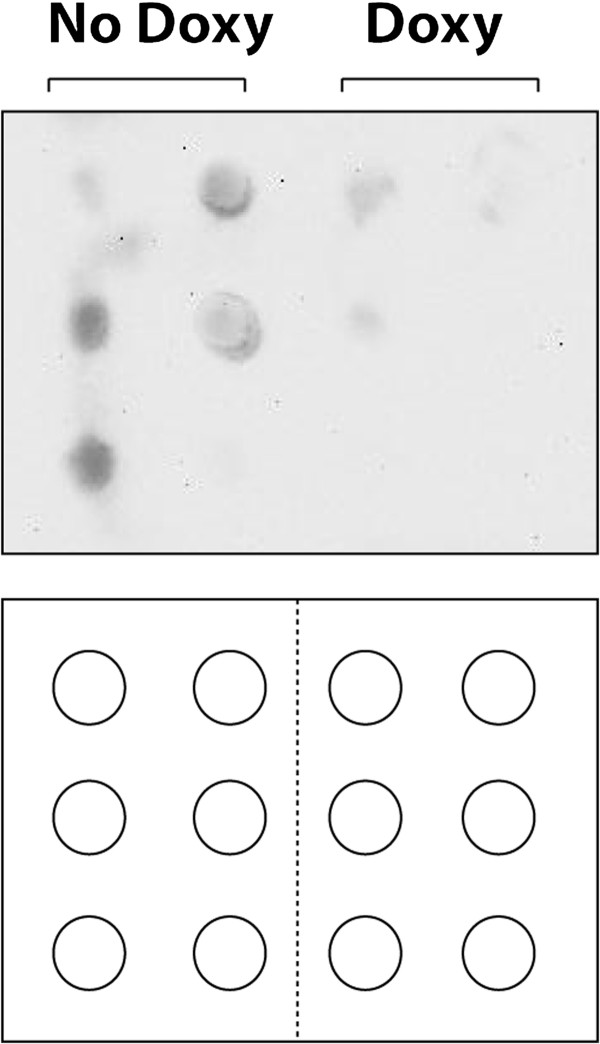
**BAL syndecan-1.** Immunoblots for syndecan-1 in mice exposed to 72 h of oral doxycycline or to no doxycycline (n = 6/group). The diagram shows the loading template, with each circle representing one well containing one sample. The signal is decreased in the mice treated with doxy.

Tetracyclines are known to be broad inhibitors of metalloproteinases, including MMP7 [9,10]. Syndecan-1 is a well-established physiological substrate of MMP7 [8,11]. Thus, we measured cleaved syndecan-1 in the BAL fluid of a subset of mice as a marker of MMP activity (Figure 6). We observed a decrease in cleaved syndecan-1 in the mice treated with doxy, suggesting decreased metalloproteinase activity (i.e. MMP7).

## Discussion

The main finding of this study was that the administration of doxycycline resulted in a significant reduction in the total number of BAL PMNs in mice challenged with LPS. Interestingly, the measurements of lung permeability in LPS-treated mice were not affected by doxycycline. Thus, doxycycline specifically inhibited PMN migration into the airspaces of the lung without affecting other parameters of lung injury.

Tetracyclines are known to be broad inhibitors of the synthesis and activity of metalloproteinases, including MMP7 (matrilysin) [9,10]. In the lungs, syndecan-1 shedding is MMP-dependent and primarily mediated by MMP7 [11,12]. Therefore, we used syndecan-1 shedding as a marker of MMP activity in the lungs in the presence or absence of doxycycline, and found that doxycycline reduced the shedding of syndecan-1 into the BAL fluid.

PMNs incubated in the presence of doxycycline exhibited similar chemotaxis as compared with PMNs incubated without doxycycline. Because KC, the murine ortholog of human IL-8 (CXCL8), is one of the most important PMN chemoattractants in mice, the *in vitro* results suggest that a defect in PMN migration or its ability to sense a chemokine gradient is an unlikely explanation for the decrease in intra-alveolar PMNs seen in the mice that received doxycycline.

An increasing number of data suggests that tetracyclines in general, and doxycycline in particular have a powerful anti-inflammatory effect. In mice, the administration of doxycycline in the water at a dose of 1.5 mg/kg is protective in a model of LPS-induced sepsis [13]. Furthermore, doxycyline at 2.0 mg/kg attenuates lung injury induced by LPS, doxycycline-resistant *S. pneumoniae* and bleomycin; and reduces inflammation in a model of asthma [4,5,14]. In humans, doxycycline has been used in the treatment of lymphangioleiomyomatosis (LAM) [15,16] and decreases proteinuria in diabetic nephropathy [17], although it was found to have no effect in osteoarthritis [18]. Clearly, doxycycline has anti-inflammatory properties that are independent of its antimicrobial activity.

Our study has relevance for scientists investigating the role of specific genes using the rtTA/tetO system. If the proper controls are in place, the rtTA/tetO system is still a powerful way to design *in vivo* experiments to test research hypotheses; however, comparing mice treated with and without doxycycline can be misleading. Instead, comparisons should be performed between double transgenic mice carrying the entire rtTA/tetO system and single transgenic mice carrying only the tetO operator, all in the presence of doxycycline.

## Conclusions

In summary, we found that doxycycline doses commonly used in the rtTA/tetO system resulted in inhibition of PMN migration into the airspaces. Investigators using the rtTA/tetO system to study mechanisms of lung injury should proceed with extreme caution and design studies with the proper controls to account for the confounding effects of doxycycline. We conclude that doxycycline is an inhibitor of PMN migration into the airspaces of the lung.

## Competing interests

The authors declare that they have no competing interests.

## Authors’ contributions

AM performed the animal experiments and participated in the drafting of the manuscript. SG performed immunoassays and flow cytometry and participated in the drafting of the manuscript. SEG performed the chemotaxis assays and participated in editing the manuscript. PC performed syndecan-1 dot blot assays and participated in the drafting of the manuscript. GMB conceived the study, participated in its design and coordination and drafted the manuscript. All the authors approved the final version of the manuscript.
